# An Adult Patient With Acute Ischemic Stroke and Carotid Stenosis Presenting to a Chiropractor: A Case Report

**DOI:** 10.7759/cureus.37209

**Published:** 2023-04-06

**Authors:** Neal B deBuhr, Robert J Trager, Cliff Tao

**Affiliations:** 1 Chiropractic Private Practice, Thrive Chiropractic, Cedar Falls, USA; 2 Chiropractic, Connor Whole Health, University Hospitals Cleveland Medical Center, Cleveland, USA; 3 Radiology, Private Practice, Chiropractic Radiology, Irvine, USA

**Keywords:** cervical spine, covid-19, klippel-feil syndrome, stroke, carotid arteries, chiropractic

## Abstract

A 59-year-old male, with a recent history of acute respiratory syndrome coronavirus 2 (SARS-CoV-2) pneumonia, presented to a chiropractor with a one-week history of numbness in the right upper and lower extremity that was triggered by neck movement, and lightheadedness/dizziness. On examination, the chiropractor noted limited, painful cervical spine range of motion, right upper extremity weakness, patellar hyperreflexia, positive Hoffman's and Trömner's signs bilaterally, nystagmus, a sluggish right pupillary light reflex, and carotid bruit. Cervical radiographs were suggestive of Klippel-Feil syndrome. The chiropractor suspected a vascular cause such as a transient ischemic attack and referred the patient to the emergency department, which the patient visited the following day. The patient was admitted, and MRI revealed multiple tiny acute to subacute cortical infarcts of the left frontal and parietal lobes while sonography demonstrated left internal carotid artery stenosis. The patient was treated with anticoagulant and antiplatelet medications and carotid endarterectomy with a positive outcome. Given the overlap between symptoms of stroke and those of the cervical spine, chiropractors should be prepared to recognize potential stroke patients and refer them for emergent medical management.

## Introduction

A stroke is defined as a sudden loss of neurological function, caused by impairment of cerebral circulation. It is the second most common cause of death in adults worldwide [1]. Symptoms of stroke vary depending on the brain region affected; common clinical features may include sudden numbness or weakness affecting the face or upper or lower extremity, sudden visual or speech disturbance, difficulty in speaking, headache, dizziness, and balance or gait disturbance [1]. Previous research has suggested that patients with stroke may inadvertently seek care from chiropractors, unaware that their symptoms are caused by an emergency vascular condition [2-4]. However, few case reports have highlighted this phenomenon.

Previous case reports have suggested that cervical spinal manipulation (a common treatment used by chiropractors) may trigger stroke by damaging or triggering embolism from the paired vertebral or carotid arteries, which run through the neck [2,3]. However, large epidemiological studies and a systematic review reported no increase in the risk of stroke following chiropractic spinal manipulation [2-4]. To explain the earlier findings of observational studies, authors have therefore suggested that individuals may present to chiropractors with a stroke in progress. According to this hypothesis, associated symptoms of stroke, such as headache or neck pain, could prompt patients to seek chiropractic care [2-4].

However, to our knowledge, there is scarce research examining how often patients with an undiagnosed stroke present to chiropractors, or why these patients seek chiropractic care. In one survey study of 2,000 chiropractors in the United States, respondents indicated encountering a patient with a stroke once per 33 years on average [5]. In contrast, a case series involving a single private chiropractic practice reported an apparently higher incidence, with three patients presenting with suspected stroke over a span of four years [6]. Another two case reports have described adult patients who presented to chiropractors who subsequently referred the patients for additional evaluation, which confirmed the presence of stroke [7,8]. In one case, the patient was a middle-aged male with head and neck pain [7], and in the other, an elderly woman with neck pain, nausea, and visual symptoms [8].

Given the limited research available on this topic, we discuss a case of an adult male with extremity numbness and dizziness presenting to a chiropractor, who suspected stroke and referred the patient for additional evaluation, which ultimately led to a positive outcome.

The patient provided written consent for the publication of this case report and any accompanying images.

## Case presentation

Patient information

A 59-year-old private pastoral care provider, with a history of Crohn’s disease, primary hypogonadism, and recent coronavirus disease 2019 (COVID-19) pneumonia six weeks previously, presented to a chiropractor in January 2022 complaining of a five-day history of progressively worsening transient episodes of “numbness and pins and needles” in the right-side extremities. His sensory symptoms were triggered by neck movement and alleviated by sitting down and rated 8/10 in severity on the numeric pain rating scale. The patient reported that these symptoms occurred four times after turning and extending his neck to the right side, causing symptoms diffusely in his right upper extremity twice, and his right lower extremity twice.

The patient was a non-smoker and did not drink alcohol. His surgical history included radiation therapy for a mediastinal seminoma at the age of 39, from which he had made a complete recovery, and right total hip replacement after sustaining a femoral fracture at the age of 47. His family history was relevant for hypertension, hyperlipidemia, and heart disease (father). The patient regularly exercised by riding his bicycle. He took prescription azathioprine (100 mg) and testosterone gel (500 mg) as well as nutritional supplements including a multivitamin, vitamin D3, quercetin, bromelain with vitamin C, omega-3 fish oil, and iron. A review of systems was relevant for a lightheadedness-type of dizziness, which was episodic and only occurred with head rotation, and noted abdominal bloating/cramping, and poor sleep. He denied any recent trauma or chest pain.

Clinical findings

On examination, the patient had a pulse rate of 72 beats per minute, a respiration rate of 18 breaths per minute, an oral temperature of 98.4 °F, a blood pressure of 142/90 mmHg, and a body mass index of 25.8 kg/m^2^. The patient demonstrated a normal gait, and heel and toe walk, and had a normal Romberg test. His active cervical range of motion was limited with flexion at 30° (normal: 50°) with pain, extension at 5° (normal: 60°) with pain, left rotation at 30° (normal: 80°) without pain, and right rotation at 20° (normal: 80°) with pain. Motor testing revealed 4/5 strength (Medical Research Council scale) of right wrist extension and finger abduction, with lower extremity strength being normal. However, when the patient’s cervical spine was in a flexed position, the muscle strength testing was normal. Muscle stretch reflexes were 2+ in the upper and lower limbs, aside from 3+ bilateral patellar reflexes. The plantar response was down going bilaterally. Hoffman's sign and Trömner's sign were present bilaterally yet normalized with cervical flexion. A sensory examination of the fingers and toes was normal, including light touch, pinprick, position sense, and vibration sense. A cranial nerve examination revealed horizontal nystagmus of both eyes when looking to the right and a sluggish right pupillary light reflex. Cervical extension-rotation reproduced the patient’s right upper extremity numbness and lightheadedness/dizziness immediately when performed on either side (right or left). There were no abnormalities with rapid alternating and fine finger movements, heel-shin testing, and finger-nose testing.

**Figure 1:**
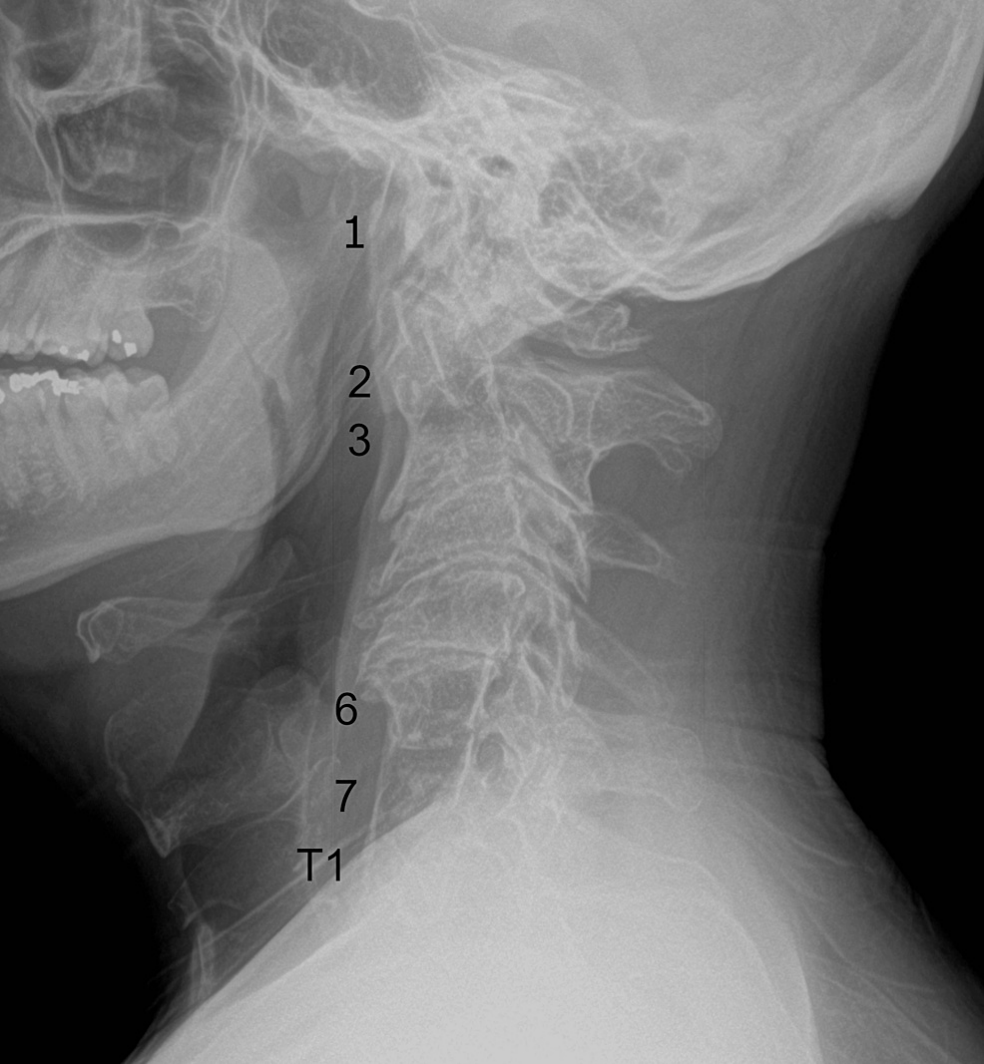
Cervical radiograph - lateral view Assimilation of the atlas is indicated as the first cervical vertebra is not completely identifiable (1). The second and third cervical vertebrae are non-segmented (2, 3). In addition, the sixth and seventh cervical vertebrae (6, 7) are non-segmented with the first thoracic vertebra (T1)

Given the presence of neurological symptoms and signs that corresponded to neck movements, limited cervical spine range of motion, upper extremity motor deficits, and hyperreflexia, the chiropractor initially considered a diagnosis of degenerative cervical myelopathy and ordered cervical spine radiographs, which were performed in-office that day. These radiographs demonstrated several areas of non-segmentation between vertebrae, consistent with Klippel-Feil syndrome (Figure 1).

The chiropractor reconsidered the differential diagnosis at this point and noted signs and symptoms that were not fully explained by the patient's radiographic findings of Klippel-Feil syndrome, such as a carotid bruit, nystagmus, and a sluggish pupillary reflex. Consequently, the chiropractor recognized the possibility of a serious vascular issue and did not proceed with any chiropractic treatments. Instead, the chiropractor considered that the patient’s symptoms could be explained by a vascular emergency such as a transient ischemic attack or cervical artery pathology. He counseled the patient, who was accompanied by his wife, on the seriousness of these conditions, and recommended that he immediately visit the emergency department. However, the patient preferred to visit his primary care provider first. The chiropractor, therefore, sent his notes to the primary care provider’s office that evening and called the provider early the following morning to inform the physician about his concerns for potential vascular pathology. That evening, the chiropractor called to check on the patient, and his wife reported that he had experienced another episode of sensory symptoms, and they had obtained an appointment with the primary care provider for the following morning.

Examination by the primary care provider revealed a left carotid systolic murmur, bilateral patellar hyperreflexia, and decreased sensation in the right V1 (forehead) and V2 (maxillary region). The provider advised the patient that his symptoms were concerning for a potential vascular etiology such as vertebral artery stenosis/dissection, carotid artery stenosis, or stroke. The provider recommended that the patient visit the emergency department, which he did immediately. The primary care provider also contacted an on-site emergency physician, recommending that the patient undergo advanced brain imaging.

**Figure 2:**
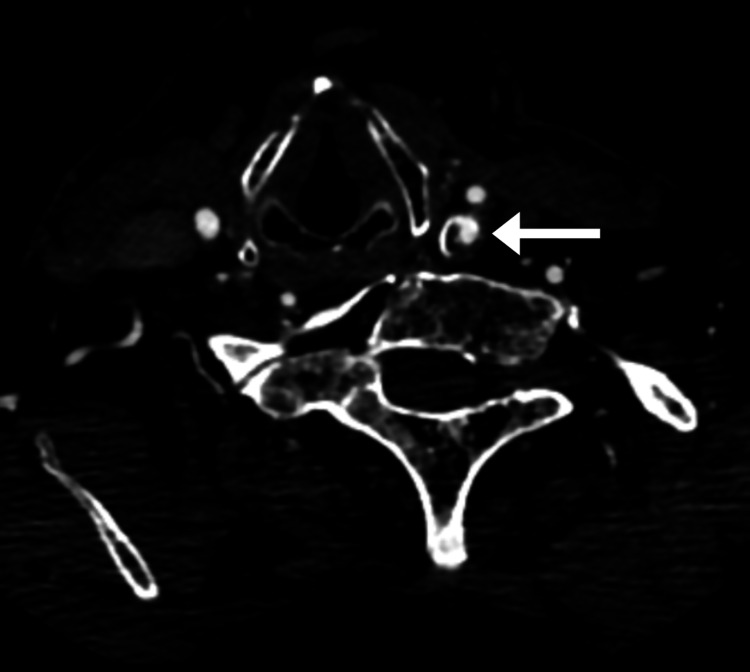
Axial CT angiography of the neck with contrast There is 68% stenosis of the proximal left internal carotid artery (arrow) CT: computed tomography

**Figure 3:**
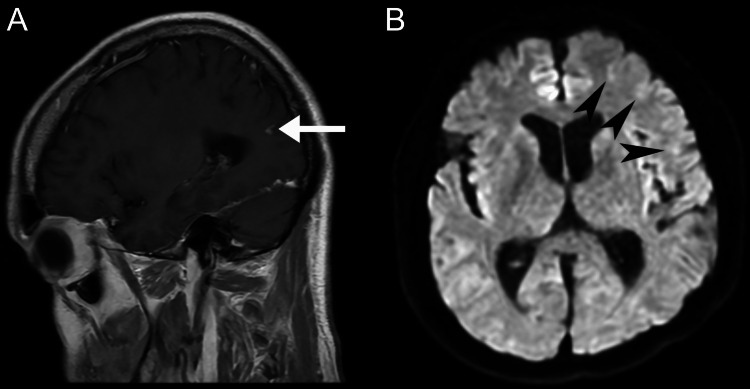
Brain MRI - sagittal T1-weighted post-contrast (A) and axial diffusion-weighted (B) images Tiny enhancing foci in the left parietal lobe cortex (arrow) and tiny acute to subacute cortical infarcts at the left frontal lobe were favored to represent enhancing tiny subacute infarcts MRI: magnetic resonance imaging

**Figure 4:**
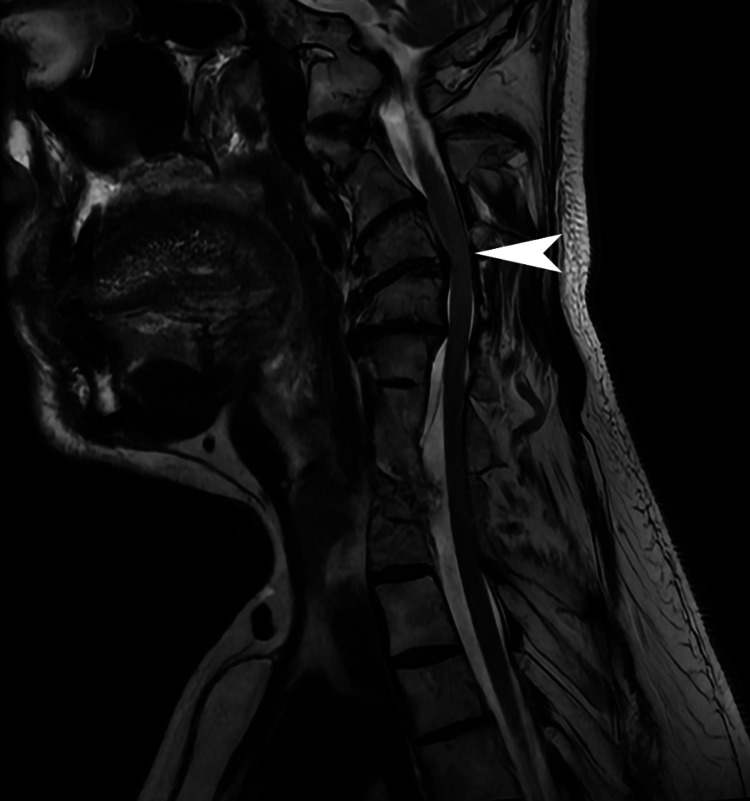
Sagittal T2-weighted cervical spine MRI There is a focal kyphosis at C5/6 and associated disc bulge causing severe canal stenosis (diameter of 7 millimeters) and mild spinal cord flattening (arrowhead). Congenital failure of segmentation is re-demonstrated with occipitalization, fusion of C2/3, and fusion of C6/7/T1 (not indicated) MRI: magnetic resonance imaging

The patient was admitted to the hospital that same day, where he underwent CT of the brain and neck with and without contrast (Figure 2), which revealed 68% stenosis of the proximal left internal carotid artery, 36% stenosis of the proximal right common carotid artery, and no acute intracranial findings or evidence of arterial dissection or aneurysm. The patient subsequently underwent an MRI of the brain (Figure 3) and cervical spine (Figure 4). Brain MRI was consistent with multiple areas of tiny acute to subacute cortical infarcts in the left frontal and parietal lobes. Cervical spine MRI was consistent with cervical spinal stenosis and, in addition, re-demonstrated the levels of non-segmentation consistent with Klippel-Feil syndrome.

During hospitalization, the patient’s electrocardiogram revealed normal sinus rhythm and his echocardiography revealed an ejection fraction of 50-55% and right ventricular systolic pressure of 25 mmHg. Sonography revealed 68% stenosis of the left internal carotid artery. The patient was prescribed apixaban (5 mg), clopidogrel (75 mg), and atorvastatin (40 mg) for stroke prophylaxis and was scheduled for carotid endarterectomy and discharged. One month later, the patient underwent a successful carotid endarterectomy.

At a follow-up visit at the neurologist a month after the endarterectomy, the patient showed considerable improvement when compared to his initial presentation, only revealing mild neck and left scapular pain and transient numbness in the hand when driving. Examination revealed normal findings with strength, sensation, reflexes, and no pathological reflexes, with the only abnormality being 3+ bilateral patellar reflexes. The neurologist considered that these mild abnormalities may be related to the patient’s cervical spinal stenosis and referred the patient to a neurosurgeon. The patient ultimately saw three different neurosurgeons over the following three months and was reportedly told that he may require surgery, but considering that he was mostly neurologically intact, he could instead be closely observed with periodic follow-up appointments and imaging. He was also advised that he needed to wait a year after the carotid endarterectomy to undergo surgery and that the Klippel-Feil syndrome could complicate any cervical spine surgery.

The chiropractor continued to communicate with the patient monthly to check on his status. All follow-up visits with other medical specialists were coordinated by the patient's other providers. One year after the initial chiropractic visit, the patient had not developed any additional cerebrovascular complications and had not undergone cervical spine surgery.

## Discussion

This report discusses a case of an adult man with extremity numbness triggered by neck movements and lightheadedness who presented to a chiropractor. The chiropractor recognized that the patient’s symptoms and neurological abnormalities could represent a serious vascular disorder and referred the patient to the emergency department. In addition, the chiropractor contacted the patient's primary care physician to alert them about the concern for potential vascular pathology, which helped expedite the patient receiving medical care. Advanced imaging revealed multiple acute to subacute cortical infarcts and internal carotid artery stenosis, confirming the chiropractor's suspicion of a vascular issue. The patient received anticoagulant and antiplatelet medications as well as carotid endarterectomy, resulting in symptom improvement. To our knowledge, this is one of only a handful of case reports describing patients with a previously undiagnosed stroke presenting to a chiropractor [6-8]. 

This report highlights that examination findings of directional preference (e.g., features that vary per spinal position) should be interpreted with caution and are not necessarily diagnostic of a degenerative spinal disorder [9]. Symptoms related to the cervical arteries may also be modified with position [10], thereby yielding a confusing picture if both spinal and vascular disorders are present. As the patient’s reflex tests normalized with cervical flexion, the chiropractor considered a diagnosis of cervical spinal stenosis. However, additional neurological findings, namely nystagmus, carotid bruit, and sluggish pupillary response, were unexplained by any potential stenosis. This case highlights that the management of vascular signs and symptoms should take priority even when other examination features are suggestive of a degenerative spinal disorder.

The patient in this case had several relevant risk factors for stroke. A recent systematic review has suggested that COVID-19 is a risk factor for ischemic stroke [11]. The authors noted that COVID-19 may cause hypercoagulation or promote thromboembolism, which may be particularly problematic for individuals with underlying vascular stenosis [11], such as the patient in the current case. In addition, one study has suggested that Klippel-Feil syndrome may be a risk factor for carotid artery damage and subsequent stroke. As several vertebrae are congenitally fused together, there is a gross loss of range of motion and potential hypermobility in the areas that are not fused, which may create an abnormal shear force on the internal carotid artery [12]. Our patient also had internal carotid artery stenosis, a risk factor for stroke [1], which was undiagnosed prior to visiting the chiropractor. However, there are several additional risk factors for stroke that were not present in the current case, such as a history of hypertension, diabetes, atrial fibrillation, hyperlipidemia, and prior stroke [1].

The symptoms reported in previous cases describing patients who presented to a chiropractor with stroke have varied. Two patients in previously published cases had neck pain, while one had a headache [7,8]. However, in the current case, neck pain was only present with movement, while headache was not present. Dizziness and extremity neurological deficits were likewise reported in previous similar cases, as well as other neurological symptoms of the head and face such as slurred speech and facial weakness [6]. Accordingly, chiropractors should recognize the variability in symptoms related to stroke.

Detection of stroke in the outpatient setting depends on a thorough history and examination. Asking the patients specific questions about a previous diagnosis of stroke or transient ischemic attack, painless weakness, numbness, loss of vision, weakness, and ability to express themselves has a high sensitivity and moderate specificity for detecting stroke [13]. A general examination that may include vital signs, mental status, gait assessment, and neurological tests of sensation, strength, reflexes (to assess for upper motor neuron signs), and the cranial nerves is also critical for identifying stroke [1,14]. Additional specialized testing such as the head impulse-nystagmus-test of skew (HINTS) test may be useful in the case of acute, continuous dizziness [1], but has not been studied in the chiropractic setting, to our knowledge.

In addition, a variety of clinical scales may aid in clinicians’ assessment of acute stroke. Screening tests that examine both cortical and motor function have optimal diagnostic accuracy [15], e.g., the Rapid Arterial oCclusion Evaluation (RACE) scale for stroke, which involves assessment for facial palsy, arm and leg motor impairment, head and gaze deviation, and hemiparesis [15]. Additional scales include the Field Assessment Stroke Triage for Emergency Destination (FAST-ED) scale and the National Institutes of Health Stroke Scale (NIHSS) [15].

Although it is not clear how often patients with an undiagnosed stroke present to chiropractors, this case highlights that such events may occur. Management of stroke is time-sensitive, and favorable outcomes can be achieved with faster access to early intervention and specialist care [15]. Therefore, chiropractors should be aware of the various risk factors and examination procedures that are used to identify a stroke. In the current case, several abnormalities on examination helped the chiropractor identify a potential vascular emergency and make the appropriate emergency referral.

This case report has certain limitations. Cervical spine radiographs may not have been immediately useful in the current case, as a referral to the emergency department for a suspected vascular condition could have been made based on the patient’s symptoms and examination findings alone. Fortunately, this step did not significantly delay care as the patient was referred for emergency care regardless. The findings from this report may not be broadly generalizable to more common cases of stroke given the patient’s unique underlying Klippel-Feil syndrome, central canal stenosis, and potential degenerative cervical myelopathy. These conditions may have confounded his clinical presentation by contributing to upper motor neuron signs. However, the cranial nerve findings, nystagmus, and the entirety of the patient’s presentation could not be solely attributed to the degenerative cervical spine findings.

## Conclusions

This report described a case of an adult man who presented to a chiropractor with neurological signs and symptoms suggestive of stroke and was appropriately referred to the emergency department, subsequently receiving medical treatment and carotid endarterectomy with a positive outcome. As stroke symptoms vary, patients may present to chiropractors unaware that they have a serious vascular disorder. Chiropractors should be able to recognize key risk factors and clinical features of a stroke to identify such patients and promptly refer them for emergency care.

## References

[1] Wallace EJ, Liberman AL (2021). Diagnostic challenges in outpatient stroke: stroke chameleons and atypical stroke syndromes. Neuropsychiatr Dis Treat.

[2] Whedon JM, Song Y, Mackenzie TA, Phillips RB, Lukovits TG, Lurie JD (2015). Risk of stroke after chiropractic spinal manipulation in medicare B beneficiaries aged 66 to 99 years with neck pain. J Manipulative Physiol Ther.

[3] Cassidy JD, Boyle E, Côté P, Hogg-Johnson S, Bondy SJ, Haldeman S (2017). Risk of carotid stroke after chiropractic care: a population-based case-crossover study. J Stroke Cerebrovasc Dis.

[4] Church EW, Sieg EP, Zalatimo O, Hussain NS, Glantz M, Harbaugh RE (2016). Systematic review and meta-analysis of chiropractic care and cervical artery dissection: no evidence for causation. Cureus.

[5] Daniel DM, Ndetan H, Rupert RL, Martinez D (2012). Self-reported recognition of undiagnosed life threatening conditions in chiropractic practice: a random survey. Chiropr Man Therap.

[6] Leach RA (2010). Patients with symptoms and signs of stroke presenting to a rural chiropractic practice. J Manipulative Physiol Ther.

[7] Kier AL, McCarthy PW (2006). Cerebrovascular accident without chiropractic manipulation: a case report. J Manipulative Physiol Ther.

[8] Liebich JM, Reinke TS (2014). Presentation of an 85-year-old woman with musculoskeletal pain to a chiropractic clinic: a case of ischemic stroke. J Chiropr Med.

[9] May S, Runge N, Aina A (2018). Centralization and directional preference: an updated systematic review with synthesis of previous evidence. Musculoskelet Sci Pract.

[10] Kranenburg HA, Tyer R, Schmitt M, Luijckx GJ, van der Schans C, Hutting N, Kerry R (2019). Effects of head and neck positions on blood flow in the vertebral, internal carotid, and intracranial arteries: a systematic review. J Orthop Sports Phys Ther.

[11] Nannoni S, de Groot R, Bell S, Markus HS (2021). Stroke in COVID-19: a systematic review and meta-analysis. Int J Stroke.

[12] Sato K, Yazawa Y, Igasaki S, Saito T, Kawabata Y, Nakashima I (2022). A case of ischemic stroke accompanied by multiple arterial dissections associated with Klippel-Feil syndrome. J Stroke Cerebrovasc Dis.

[13] Sung VW, Johnson N, Granstaff US, Jones WJ, Meschia JF, Williams LS, Safford MM (2011). Sensitivity and specificity of stroke symptom questions to detect stroke or transient ischemic attack. Neuroepidemiology.

[14] Sun Z, Yue Y, Leung CC, Chan MT, Gelb AW (2016). Clinical diagnostic tools for screening of perioperative stroke in general surgery: a systematic review. Br J Anaesth.

[15] Antipova D, Eadie L, Macaden A, Wilson P (2019). Diagnostic accuracy of clinical tools for assessment of acute stroke: a systematic review. BMC Emerg Med.

